# Computational Analysis of Distance Operators for the Iterative Closest Point Algorithm

**DOI:** 10.1371/journal.pone.0164694

**Published:** 2016-10-21

**Authors:** Higinio Mora, Jerónimo M. Mora-Pascual, Alberto García-García, Pablo Martínez-González

**Affiliations:** Specialized Processor Architecture Laboratory, Department of Computer Technology and Computation, University of Alicante, Alicante, Spain; Chongqing University, CHINA

## Abstract

The Iterative Closest Point (ICP) algorithm is currently one of the most popular methods for rigid registration so that it has become the standard in the Robotics and Computer Vision communities. Many applications take advantage of it to align 2D/3D surfaces due to its popularity and simplicity. Nevertheless, some of its phases present a high computational cost thus rendering impossible some of its applications. In this work, it is proposed an efficient approach for the matching phase of the Iterative Closest Point algorithm. This stage is the main bottleneck of that method so that any efficiency improvement has a great positive impact on the performance of the algorithm. The proposal consists in using low computational cost point-to-point distance metrics instead of classic Euclidean one. The candidates analysed are the Chebyshev and Manhattan distance metrics due to their simpler formulation. The experiments carried out have validated the performance, robustness and quality of the proposal. Different experimental cases and configurations have been set up including a heterogeneous set of 3D figures, several scenarios with partial data and random noise. The results prove that an average speed up of 14% can be obtained while preserving the convergence properties of the algorithm and the quality of the final results.

## Introduction

The advance of the computational sciences is heading to the development of applications and technologies oriented towards improving the quality of life of the citizens. In this context, the automatic knowledge of the world surrounding us provide information for offering valuable services [1, 2]. The new application environments such as internet of things and mobile computation demand the development of methods able to be used in more restrictive scenarios.

Shape registration is a key stage in the process of reconstruction or acquisition of 3D surfaces so that it is considered a cornerstone in the fields of computer vision and graphics. It is also a task of major importance in many other fields like biometric applications [3], 3D model reconstruction from multiple range images [4], medical diagnostic support tools and computer aided interventions [5], quality control of manufactured pieces [6], and robotic applications ranging from full environment reconstruction for navigation to particular objects 3D model creation and tracking [7].

Nowadays, range sensors obtain depth information so that we can capture 3D datasets from different points of view, each one of them represented using a particular coordinate system. A lot of applications like the ones mentioned before require a full or partial scene reconstruction from the data provided by the sensors over different points of view. In order to reconstruct the surfaces or shapes of the original scene, we have to combine the different datasets with their own coordinate systems in a process called “shape registration”.

The goal of shape registration is the transformation of different 3D datasets to represent them in one common coordinate system so that those elements that overlap in both sets are properly aligned allowing the reconstruction of the original surfaces. The registration process may be applied to rigid or non-rigid shapes. In the case of rigid shapes, the transformations which aligns both surfaces are rigid too (rotation and translation) so that the solution space is bounded to six degrees of freedom. On the other hand, non-rigid shape requires a non-rigid transformation which takes into account the possibility of deformation so that the solution space is increased considerably.

Registration can be classified according to its granularity, distinguishing coarse and fine grained methods. The objective of coarse grained registration is to obtain a quick estimate of the transformation to roughly align both shapes while fine grained techniques use that initial estimate to refine it iteratively in order to find the best alignment in terms of precision under a set of restrictions.

Currently, the most popular method for 3D rigid registration for the Robotics community is the Iterative Closest Point (ICP) algorithm [8]. This is a fine grained registration method characterized by its simplicity and effectiveness. A wide set of applications of different fields make use of this algorithm in order to compute rigid registrations, however, the algorithm has a high computational complexity (quadratic with respect to the number of points in its original variant) which renders impossible or at least makes difficult certain applications which require the processing of high density point sets provided by high precision sensors.

The search for solutions to overcome this problem is the main motivation of this research. Many variants have been proposed in the literature to improve the algorithm’s performance, either by reducing the number of points or by decreasing the needed iterations or even reducing the complexity of its most expensive phase in terms of computing resources: the search of nearest neighbours. Nevertheless, despite reducing its complexity, in many cases those variants tend to have a negative impact on precision or even on the convergence domain, limiting the possible application scenarios. Hence, the general objective of this research is to find ways to improve the performance of the algorithm without affecting the quality nor reducing its possible application scenarios.

The main contribution of this work is a general improvement for the algorithm, carried out by an interdisciplinary research based on the fusion of mathematical and geometric concepts, such as distance metrics, with its computational component by taking into account their associated operative cost and their impact on the algorithm's execution time. The proposed modification of the distance metric is focused on improving the low-level computing performance at the hardware layer of the system architecture. The focus is to design a distance as simple as possible. That is, that use the fewer number of mathematical arithmetic functions with the lower costs. The novelty of the proposal lies in easing the computational effort of the ICP algorithm by fitting the calculations to the arithmetic hardware of the architecture. In this way, a better adaptation of the methods to the hardware implies a better performance [9]. In addition, computer arithmetic techniques such as pre-calculated results [10] and imprecise computation paradigm [11] could be used in order to further improving the performance.

The working hypothesis of the research consist on speeding up the mathematical distance function in which the ICP is based without perceptible effects on quality and robustness. To achieve this goal, this work analyses other distance metrics whose computational cost is reduced respect to the Euclidean one and evaluates the algorithm's convergence properties as well as the registration quality in terms of convergence domain and final registration error.

The rest of this paper is structured as follows: Section 2 provides a general view of the most remarkable variants of the ICP algorithm published, focusing on those whose goal is to accelerate the method; in addition, those elements which differentiate our proposal are outlined; section 3 introduces the ICP algorithm and the proposed distance metrics; Section 4 describes the experiments that will be carried out; next, Section 5 discusses the main results of the experiment; at last, Section 8 concludes this work with an overview of the results and the accomplished goals to end the paper putting this work in context with other proposals and enumerating some future work possibilities.

## Related Work

This section discusses the state-of-the-art of ICP algorithm. It is not intended to be exhaustive but to show the most remarkable works as representative of the intensive research on this topic.

The research efforts are mainly focused on designing variants of the algorithm with specific features for certain applications and/or increasing the performance by improving the stages of the calculation method. The ICP algorithm can be divided into six stages [12]: point selection, matching, pair weighting, outlier removal, error metric, and minimization. The next subsections review the previous works and the relevant proposals of the algorithm for each one of its stages, focusing on matching and error metric because our proposal will directly impact on them.

The Table 1 provides a summary of the contributions outlined in this review which has been organized according to the part of the algorithm addressed. Finally, a findings subsection is added which describes our contributions to previous work.

**Table 1:** Main contributions outlined.

Problem addressed	Research works
Rigid Registration issue and ICP algorithm	Surface registration techniques [13] Image registration methods [14] Registration of 3D point clouds [15] Shape correspondence [16] Efficient variants of the ICP algorithm [17] Accurate free form shape matching [18] Probabilistic registration [19] Non-rigid registration [20, 21] Inclusion of *a priori* knowledge [22] Using Kd-trees [23] Using closest points caching [24] Binary space-partitioning trees [25] Hardware implementations on CPU [26] and GPU [27]
Point selection	Uniform subsampling [28] Random selection [29] Selection based on color or intensity information [30] Normal-space sampling [12]
Matching	Inclusion of additional properties [30, 31] Taking into account different types of noise [32]
Pair weighting	Weights based on normal colors [33] Weights based on compatibility criteria
Outlier removal	Rejection of a percentage of the worst correspondences [34] Rejection of pairs over a certain threshold [12] Rejection of pair in the mesh boundaries [9] Rejection of pairs which aren't consistent neighbours [35]
Error metric	Point-to-point metric [8] Point-to-plane metric [36]
Minimization	Unitary quaternions method [37] Singular Value Decomposition (SVD), orthonormal matrices or double quaternions [38] Gradient descent, annealing or physical system simulations [39]

### Rigid Registration issue and ICP algorithm

There are several review works relating to the general rigid registration issue [13–16] and works focused specifically on to the study of the ICP algorithm as a whole and its different versions [17, 18].

Among the different variants of the algorithm, it is worth highlighting the probabilistic registration for high accuracy alignment [19], the extension of the ICP algorithm for non-rigid registration [20, 21] and the variants focused on the inclusion of *a priori* knowledge to improve registration [22]. As we previously noted, one of the main problems of the algorithm is its high complexity, quadratic with respect to the number of points, because of the need of computing the distances of all points of one point cloud to all points of the other in order to obtain the closest point. In that sense, a lot of variants have directed their efforts toward the reduction of that complexity by using k-dimensional trees [23], closest points caching [24], k-means binary space-partitioning trees [25], or even hardware parallel implementations on CPU [26] or GPU [27].

### Point selection

There are variants on point selection performing a uniform subsampling [28], random selection [29], based on color or intensity information [30] or even normal-space sampling [12]. These methods have been validated as a simple and effective way of accelerating the algorithm. Our proposal introduces no change during this phase, but all the previously mentioned improvements may be coupled with ours without any problems.

### Matching

Some variants have focused their contribution on the inclusion of additional properties in the Euclidean distance metric used to make the point correspondences. For example, by adding information about color [30] or surface normals [31]. Other contributions adapted the algorithm to take into account anisotropic and heterogeneous noise [32] in which the Euclidean metric was replaced by the *Mahalanobis distance*, proving the possibility of exchanging the distance metrics. Our proposal introduces changes it this stage, the Euclidean distance metric is replaced by other metrics to reduce the computational cost.

### Pair weighting

Various implementations include some kind of weight to each pair of corresponding points in order to improve the robustness of the algorithms when facing certain situations. The most popular weighting schemas try to assign weights based on normal colors, point-to-point distance [33] and compatibility criteria [12]. In general, these methods do not affect speed but they improve the quality for certain scenarios. Our proposal does not change this stage. However, as before, these improvements can work together with in a fully compatible manner.

### Outlier removal

In order to solve the presence of statistical outliers multiple variants have been proposed with the objective of rejecting these negative correspondences: rejection of a percentage of the worst correspondences [34], rejection of pairs whose distance is over a certain threshold [12], rejection of pair in the mesh boundaries [28] or even rejection of pairs which aren't consistent with their neighbours [17]. Again, our method makes no improvement in this stage since our main goal is not the robustness of the method but its speed. However, we can always use other variants of this stage together with our proposal.

### Error metric

There are two main error metrics that are profoundly tested and widely used: the point-to-point metric [8] and the point-to-plane one [36]. The point-to-point distance consists of the summation of the quadratic distances between the points of the model and source point clouds. On the other hand, the point-to-plane distance takes into account the distance between the points of the source to the tangent planes in which the model points are. These basic error metrics can be modified to take into account other variants of the original algorithm to improve its robustness. In fact, many of the previously mentioned variants apply changes over the error metric. In our case, it is implemented the original point-to-point error metric using the same distance metric as used in the matching stage to ensure the consistency of the algorithm. That is, the Euclidean or the proposed low-cost distance metric in each case.

### Minimization

The next step is the minimization of the function or error metric previously selected. In the case of the original metric [8] there are multiple closed-form solutions: the unitary quaternions method [37], SVD decomposition, ortonormal matrices or double quaternions [38]. In addition, there are many non-closed-form solutions based on gradient descent, simulated annealing or physical system simulations [39]. These works compare different strategies for the minimization of the point-to-point metric and states that all of them achieve similar results regarding speed and precision since they all converge in linear time and offer floating point precision [38]. On the other hand, the use of a point-to-plane metric [36] usually requires the use of least squares non-linear methods for minimizing the error function.

### Findings

Many variants of the ICP algorithm have been proposed affecting all phases of the method with the objective of reducing their computational cost and the convergence time. There are proposals oriented to improve the overall algorithm (for example probabilistic [19], KD-trees [23], uniform subsampling [28] or random selection [29]) and others designed for specific application scenarios (for example, using a priori [22] or color information [30, 33] were that information are available).

In this work, it is described a straight-forward contribution to the algorithm by using low computational cost distance metric. The proposed operations are built from simple arithmetic functions of the instruction set of the standard computer architecture. This approach complements the previous works and should be applicable to the other variants of the ICP algorithm reported since they all are using distance metrics. In the next section, the proposed distance metrics are studied from an analytical point of view.

## Distance Operator Methods

### The ICP algorithm

Rigid registration can be formulated as an optimization problem with certain restriction whose objective is the alignment of surfaces or 3D data.

Let M and D be two point sets of n dimensions with cardinality of N_M_ and N_D_ respectively. Set M is commonly referred as *model* and D as *data*. The objective is to align the *data* point cloud with the *model* one, in other words, obtain the rigid transformation Φ which minimizes the mean square error between the model and the data points once the transformation (a rotation R and a translation T) is applied to the source point set D. The objective function that has to be minimized is:

$$f\left(R,\;T\right)=\;\frac{1}{N_{D}}\sum_{i=1}^{N_{D}}{\left|\left|m_{i}-R\left(d_{i}\right)-T\right|\right|}^{2}$$ (1)

where m_i_ is each point of the model set (M) and d_i_ is each point of the data set (D).

The ICP algorithm is one of the most popular and widely used methods for performing rigid registration and solve Eq (1). Its functioning is based on the closest point criteria used for establishing the correspondences, so that the corresponding point for a source point is its closest one in the model [8]. The distances between the points are calculated by using the Euclidean distance metric to define the closest point operator.

Given two 3D-points p_1_ and p_2_, the Euclidean distance between both of them d(p_1_, p_2_) is the length of the segment which connects them ||p_2_ –p_1_||. Given a point p and a point set A (with n individual points), we define the Euclidean distance of the point to the set d(p,A) as the minimum of the distances of p to each one of the points of the set A, in other words, *d*(*p*, *A*) = min_*a*∈*A*_ *d*(*p*, *a*). So that, the function which obtains the closest point to p in the point set A can be defined as follows:

$$fc\left(p,A\right)=arg\;{\text{min}}_{a\in A}\;d\left(p,a\right)$$ (2)

The algorithm sets a correspondence between each point d_i_ of the *source* point set D and the closest point in the model which will be named *y*_*i*_ ∈ *Y*, forming the set of closest points to D. From this statement we deduce that *Y* ⊆ *M*, *y* ∈ *M* and *N*_*Y*_ = *N*_*D*_.

The closest point operator C which produces the point set *Y* = *C*(*D*, *M*) in which each point y_i_ is the closest point in the model to the point d_i_ to the source point set.

$$C\left(D,\;M\right)={\left\{y_{i}=c\left(d_{i},M\right)\right\}}_{i=1}^{N_{D}}$$ (3)

Assuming this closest point criteria, the algorithm ensures the convergence if the initial position of the source point set is close enough to the model set position. Given that, in general, the correspondences obtained using the closest point operator are not the right nor the best ones from the beginning, the ICP algorithm performs an iterative refinement process. Each one of the iterations comprises three main phases: Correspondences or matching, Transformation calculation or minimization, and Update transformation or apply it.

These phases are repeated until a certain stop criterion, such as a limit for the number of iterations or a threshold for the difference of final registration error of the current iteration and the previous one, so that the algorithm stops if the transformation has enough refinement. By using this process, the algorithm's convergence is stated in the following theorem [8]: the ICP algorithm always converges monotonically to a local minimum with respect to the objective mean squared distance error function in Eq (1).

The widely distance operator used on the matching stage is the *Euclidean distance metric*. In this work, other metrics are analysed in order to achieve lower computational cost while provide similar quality as the Euclidean one. The candidate metrics are *Chebyshev* and *Manhattan* distance metrics since we expect an inferior computational cost from them due to their simplest formulations.

### Distance metrics formulation

The distance operators meet the mathematical distance conditions stated below. In this way, the registration process generically reduces the average distance between corresponding points during each iteration and the closest point determination generically reduces the distance for each point individually [8]. Therefore, the convergence theorem remains valid for them.

A distance metric *d* is defined as a function in a set X so that $d:X\times X\to {\mathbb{R}}^{+}\cup \left\{0\right\}$, being ℝ the set of real numbers. This function describes the distance between points, for example *x*, *y*, *z*, of the *X* set. Furthermore, it must meet the following conditions:

1. *d*(*x*, *y*) + *d*(*y*, *z*) ≥ *d*(*x*, *z*)
2. *d*(*x*, *y*) = *d*(*y*, *x*)
3. *d*(*x*, *x*) = 0
4. *d*(*x*, *y*) = 0 ⟹ *x* = *y*

Once the concept of distance metric has been defined, we can address the formulation of the proposed distance metrics as candidates for the reduction of the computational cost.

#### Original Euclidean distance

The Euclidean distance between the points *x* and *y* is defined as the length of the segment which connects both of them. In Cartesian coordinates, if x = (x_1_, x_2_, …, x_n_) and y = (y_1_, y_2_, …, y_n_) are two points in a Euclidean n-space, then the distance d(x, y) is determined by Eq (4).

$$d\left(x,y\right)=\sqrt{\sum_{i=1}^{n}{\left(x_{i}-y_{i}\right)}^{2}}$$ (4)

In terms of computational cost, this metric requires the calculation of *n* multiplications, *2n-1* additions/subtractions and a *square root* to obtain the distance between two *n*-dimensional points.

#### Chebyshev distance

The Chebyshev distance between the points *x* and *y* is defined as the maximum of the absolute values of the differences between their coordinates. In this way, the Chebyshev distance of two *n*-dimensional points is described by Eq (5).

$$d\left(x,y\right)=\text{max}(\left|x_{1}-y_{1}\left|,\;|x_{2}-y_{2}\right|,\ldots ,\right|x_{n}-y_{n}|)$$ (5)

The Chebyshev metric requires *n* subtractions, *n-1* comparisons to obtain the distance between two *n*-dimensional points. Since a comparison operation has similar cost than a subtraction, the whole computational cost is *2n-1* subtractions. Please, note that the absolute values calculation for real numbers implies no computation cost where real numbers are represented with sign-bit [40, 41].

#### Manhattan distance

The Manhattan distance between the points *x* and *y* is defined as the sum of the absolute values of the differences of their coordinates. Then the Manhattan distance of two *n*-dimensional points is described by Eq (6).

$$d\left(x,y\right)=\sum_{i=1}^{n}\left|x_{i}-y_{i}\right|$$ (6)

The operative cost of this metric is *2n-1* sums/subtraction and *n* absolute values to obtain the distance between two *n*-dimensional points *x* and *y*.

## Experimentation and Validation of Topological Spaces

This section aims to validate the topological spaces proposed on this work. In first place, it is analyzed the computational cost of the distance metric functions itself. Next, the experiments carried out test the ICP algorithm using the different low-cost distance metrics in it.

### Computational cost comparison of distance metrics

The analytic analysis of the formulation of Chebyshev and Manhattan metrics results in lower and simpler arithmetic operations than the Euclidean one. In this subsection, the experiments are oriented to validate empirically this finding. Thus, it is conducted a set of empirical tests to determine the speedup that may be expected by using one metric instead of another.

The experimental setup of this part is the following:

- A typical platform based on the x86 architecture has been used with the operating system Debian 7.1, 64-bits version.
- The different distance metrics have been codified in C++ in order to perform a low-level implementation of the functions.
- Series of 10^8^ distance calculations of randomly generated 3D points have been performed for each metric. The average cost is obtained for each series.

The results of the benchmark are shown on Table 2 together with the speedups obtained over the Euclidean metric.

**Table 2:** Computational cost comparison respect to Euclidean metric.

	Euclidean	Chebyshev	Manhattan
**Execution time**	0%	-20%	-43%
**Speedup**	1.00	1.247	1.766

The results shown in Table 2 confirm our operative cost analysis that we performed in previous subsections; it is a remarkable fact that the Manhattan distance is clearly better than the Chebyshev one, while its operative complexity is quite similar; this happens because of the cost of the call to the *maximum* function which is higher than performing a simple addition or subtraction that requires no additional logic.

The obtained speedup may vary depending on the used architecture and the processor family that implements the instruction set, but we do not expect a significant deviation in the results given that most current processors implement similar features and the algorithms for computing the arithmetic operations are highly optimized for the target platform.

In addition, we would like to note that we can't expect to obtain an improvement of a 20% or a 43% in the execution time of the algorithm just by simply applying the Chebyshev or Manhattan distance metrics because this change would only accelerate the matching phase which is just a fraction of the overall computation that is performed by the algorithm.

### Experimental Design and Setup

This subsection describes the exhaustive set of experiments to prove the viability of the use of the candidate metrics and their impact on the results of the algorithm. The experimentation is focused on testing the convergence, the accuracy, the robustness and the performance of using the analyzed distance metrics in the ICP algorithm.

- The convergence is the ability of the algorithm to match the Model with the Data datasets. The convergence is achieved when the algorithm ends under the stop criterion and the final Root-Mean-Square (RMS) is smaller than the threshold (ε) established.
- The accuracy is the degree of similarity between the Model and the Data once the algorithm has been completed. This is measured by the final error defined as the RMS of the last iteration of the method.
- The robustness is a measure of how the algorithm works with noisy and partial data.
- The performance is the time cost of the algorithm. It is measured in seconds, although this result is highly dependent of the experimentation hardware.

These aspects are interrelated, so that, each experiment produces outputs for all of them. However, in order to clarify the results, the experiments have been organized in three sets depending on the degree of changes made on the figures: full figures, partial figures and noise figures.

**Fig 1:**
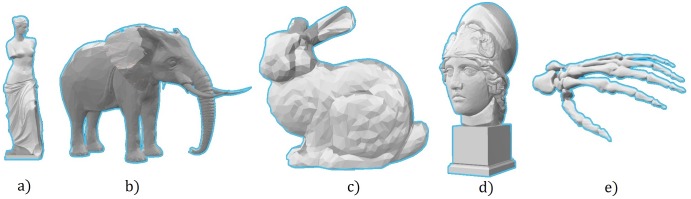
3D models. a) Venus (5688 points); b) Elephant (5132 points); c) Bunny (1494 points); d) Athenea (4721 points); e) Skeleton hand (1055 points).

The following features define the general conditions of the experimentation:

- The experiments of the algorithm have been performed in a homogeneous working environment. The operating system
- It is used a x86 architecture with Debian 7.1, 64-bits as the operating system and MathWorks Matlab^®^ 2015a as development platform.
- It is used the original implementation of ICP algorithm from Besl and McKay [8] with the needed modifications to include the different metrics and the SVD decomposition for rotation and translation estimation due to its effectiveness.
- Each test for the three distance metrics (Euclidean, Manhattan and Chebyshev) has been performed one hundred times for each 3D model. It is taken the average time of all executions, discarding values with a deviation of 20% from the median of each model in order to avoid noise due to system overloads while executing background processes.
- The registration will stop when the registration has been stabilized. That is, when the difference between the registration error in the current iteration and the one from the previous iteration is less than a threshold ε. A common value for ε is 0.05. The error of an iteration is computed as the RMS of the data set and the correspondences.
  $$e_{Iteration}=\frac{1}{N_{s}}\sum_{i=1}^{N_{s}}{\left|\left|m_{i}-d_{i}\right|\right|}^{2}$$
  where m_i_ is each point of the model set (M) and d_i_ is each point of the data set (D).
  This stop criterion is not based on the error with respect the model, instead it is based on the error of an iteration with respect the previous one.
- The set of figures used is a representative sample of the different surfaces and scenarios where 3D rigid registration techniques are often used. This set consists of five heterogeneous 3D models with different forms and sizes selected from the *Communication and Multimedia Laboratory* of the *National Taiwan University* (http://graphics.csie.ntu.edu.tw/~robin/courses/cg04/model/index.html). These models are shown in Fig 1. The number of 3D points of each full dataset is reported. These figures have been scaled to homogeneous sizes in order to obtain comparable results of the accuracy and the convergence trend.

### Full figures

This subsection tests the behavior of the ICP using the three distance metrics and the set of figures depicted in Fig 1. All the Model and Data datasets have been used with the full sets of 3D points, that is, they both have exactly the same point set.

**Fig 2:**
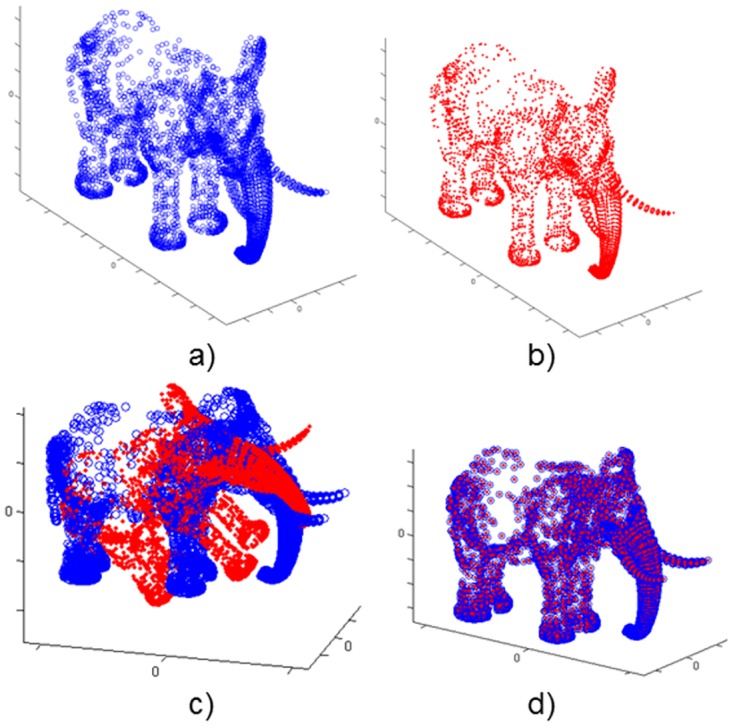
Elephant model. a) Model set; b) Data set; c) Model & Transformed Data; d) Model & Transformed Data after registration.

**Fig 3:**
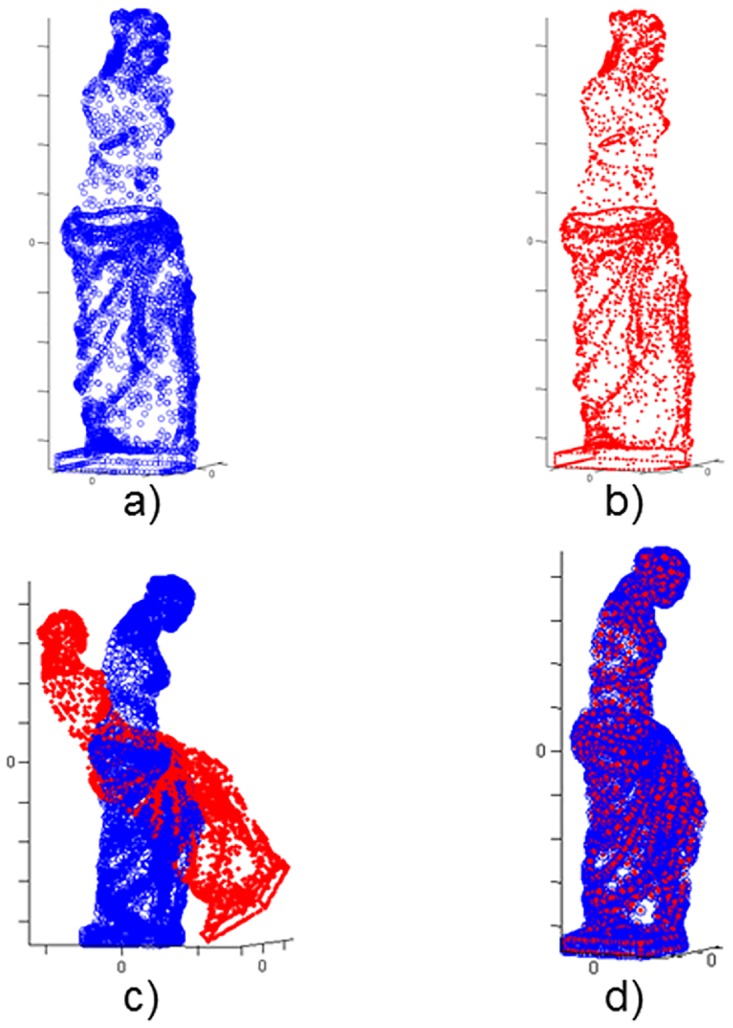
Venus model. a) Model set; b) Data set; c) Model & Transformed Data; d) Model & Transformed Data after registration.

Each test tries to register a point set into another one called model; for each scenario, the Data set has been initially transformed applying a rotation of R0(30°, 20°, 15°) and a translation of T0(0.12m, -0.08m, 0.1m) to the surface. It is a simple situation for the algorithm and it is not very useful from a practical point of view, although it allows us to evaluate the difference among metrics in a basic scenario. The Figs 2 and 3 show the experiments performed with two full models as representative case.

The experimentation results for the full scenarios are shown in Table 3. The execution time and the number of iterations have been reported. Regarding execution time, that Chebyshev distance has worse performance than Euclidean one, meanwhile Manhattan distance offers a remarkable improvement in comparison with this last distance. Regarding final registration error, all of them show similar results so neither Chebyshev nor Manhattan get significantly worse quality registrations than the one obtained with the Euclidean distance, except for the case without noise using Chebyshev.

**Table 3:** Average full figures results.

Euclidean			Chebyshev			Manhattan		
ΔTime	Iter.	Error	ΔTime	Iter.	Error	ΔTime	Iter.	Error
0%	19	8.2e-7	+87%	31	1.4e-5	-13%	19	7.9e-7

**Fig 4:**
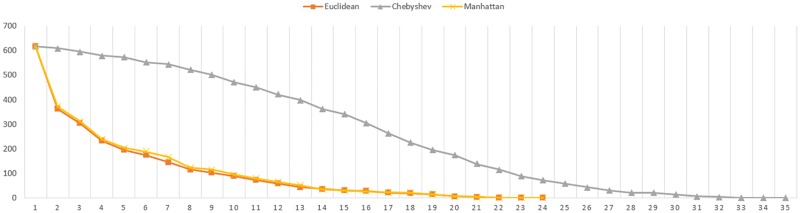
Convergence evolution for full Elephant figure.

As shown in previous table, the Chebyshev distance needs more iterations to reach convergence. The Fig 4 shows the convergence evolution of the registration for the elephant figure.

Note that number of iterations is not relevant for the conclusions of this research. Fewer iterations does not mean lower computational costs since the cost by iteration of each distance metric is not the same.

### Partial figures

When an acquisition device is registering a figure, the standard situation is taking a partial view of it. Thus, this subsection considers more realistic scenes taking part of the cloud set of the models for test the distance metrics. Two cases are considered: take a 60% of the figure and only a 30% of it.

**Fig 5:**
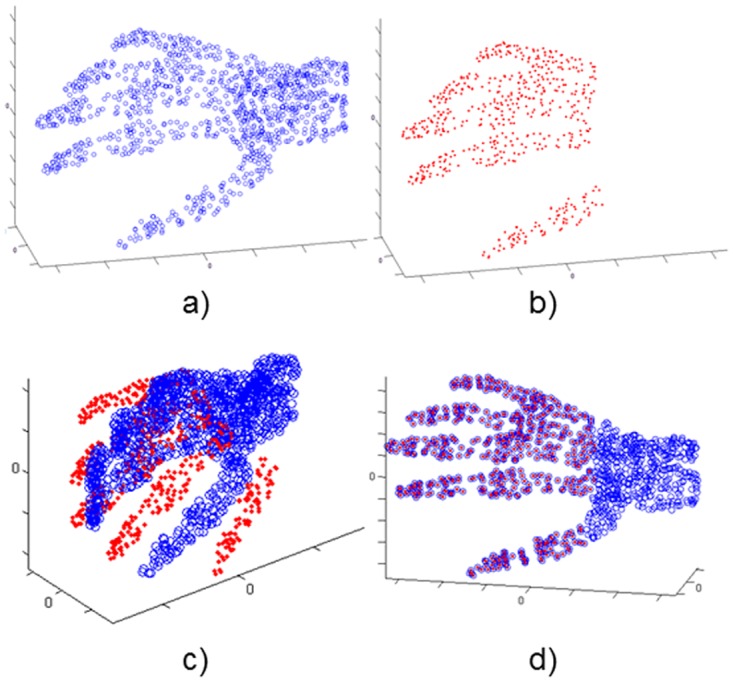
Partial figure 60%. Skeleton hand model. a) Model set; b) Data set 60%; c) Model & Transformed Data; d) Model & Transformed Data after registration.

**Fig 6:**
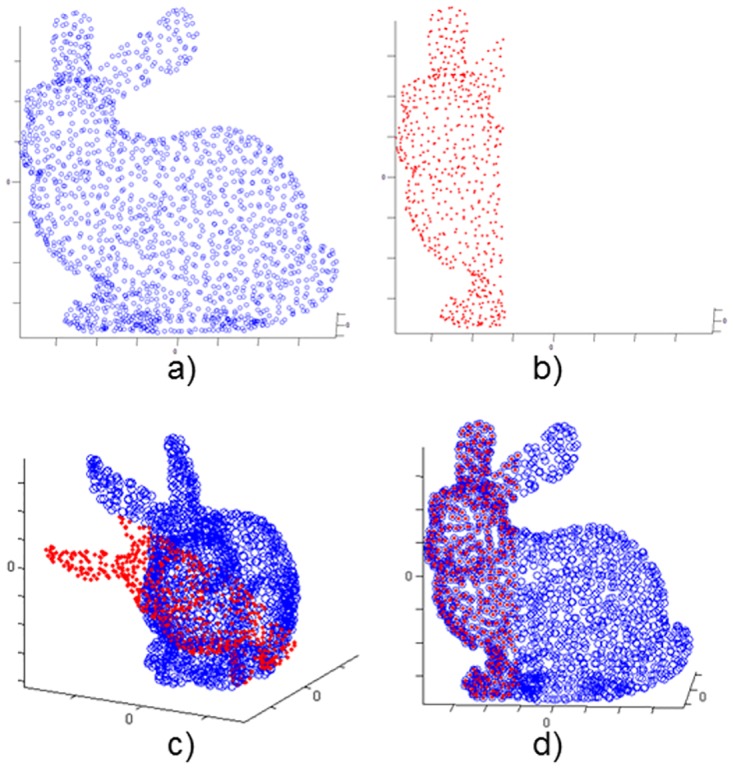
Partial figure 30%. Bunny model. a) Model set; b) Data set 30%; c) Model & Transformed Data; d) Model & Transformed Data after registration.

In these cases, the Data figure has fewer 3D points that the Model one. The 3D points discarded correspond with the 40% (or 70%) of the points of the coordinate axis where is centered the figure. The datasets have been initially transformed applying a rotation and a translation similar to previous case. For example, the Figs 5 and 6 depicts successful registration examples of the experiments for each case.

The results for these scenarios are shown in Table 4. These follow the same trend as the previously exposed data. However, in this case it is remarkable that Manhattan metric has different behaviour. The partial data affects the performance of that metric and in these cases is more similar to the Euclidean one. In contrast, the Chebyshev metric keeps providing significantly worse performance.

**Table 4:** Average partial figures results.

Partial figure	Euclidean			Chebyshev			Manhattan		
	ΔTime	Iter.	Error	ΔTime	Iter.	Error	ΔTime	Iter.	Error
60%	0%	25	2.7e-5	+77%	37	2.1e-5	-14%	25	2.4e-5
30%	0%	40	2.3e-5	+85%	69	1.6e-5	-15%	36	4.9e-7

**Fig 7:**
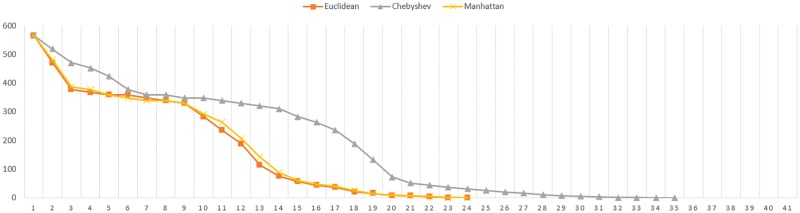
Convergence evolution for partial Skeleton Hand figure.

**Fig 8:**
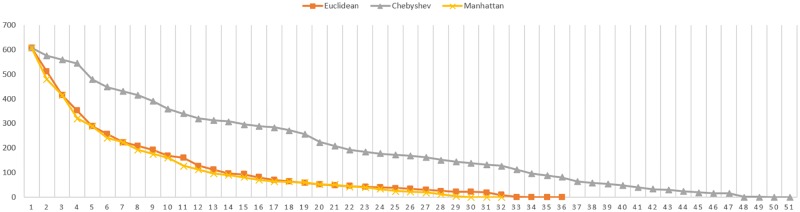
Convergence evolution for partial Bunny figure.

Regarding to the number of iterations needed, in first place, it is noted that this number increases according the registered data decreases. Secondly, the Manhattan distance seems to need less iterations than Euclidean to reach the convergence. In addition, it is proved that in these cases, the Chebyshev distance also needs more iterations to reach the convergence. The Figs 7 and 8 represents the convergence evolution of the registration shown by Figs 5 and 6 for the Skeleton Hand and Bunny models respectively.

### Noise figures

In this subsection the experiments performed are mainly focused on the robustness of the proposed distance metrics. This is quite common on the application cases of the ICP algorithm, where de acquisition devices can add some error to the measures. In these tests, it is specified two cases of noise situations: noise applied only to the Data set, and noise applied to both Model and Data sets. The datasets have been used with the full sets of 3D points and they have been initially transformed applying the same rotation and translation as in the previous cases.

**Fig 9:**
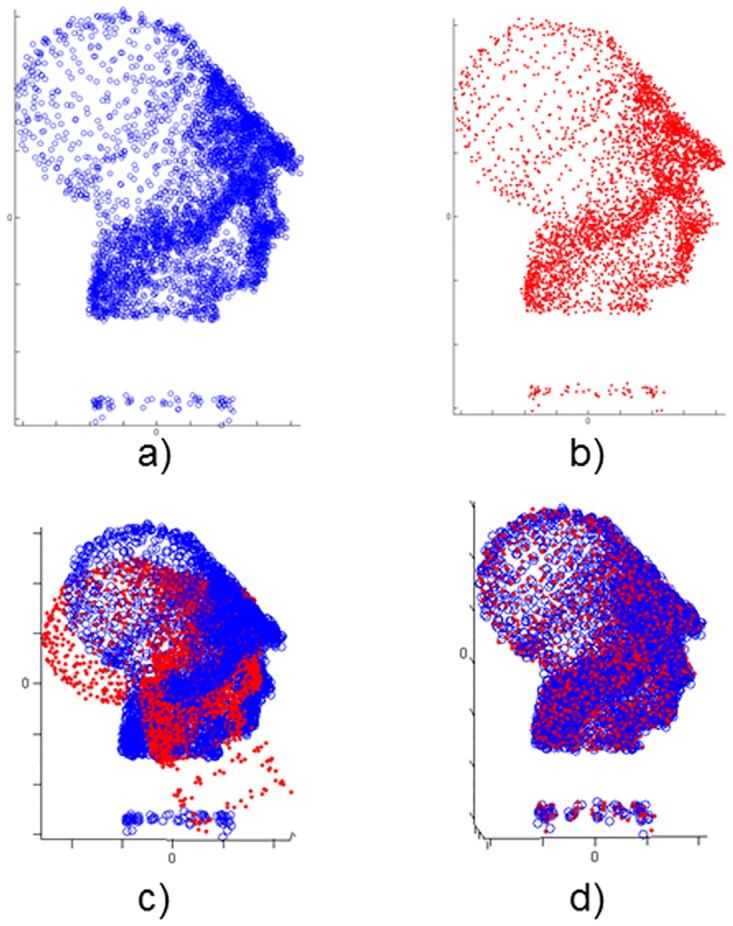
Noise Athenea figure. a) Model set; b) Data set; c) Model & Transformed Data; d) Model & Transformed Data after registration.

The applied noise consists on the application of random displacements to all coordinates of all the points of the set. The displacements have been generated from a normally distributed function with a standard deviation of 2% of the dimensions of each image. It is used the “randn” Matlab function (http://es.mathworks.com/help/matlab/ref/randn.html). The Fig 9 shows the experiments made with the Athenea model and the noise applied to both datasets.

All the cases tested reach the convergence using the three distance metrics. The results are shown in Table 5.

**Table 5:** Average full noise results.

Noise	Euclidean			Chebyshev			Manhattan		
	ΔTime	Iter.	Error	ΔTime	Iter.	Error	ΔTime	Iter.	Error
Model & Data	0%	34	44	+89%	60	43	-19%	29	45
Data	0%	23	50	+81%	39	50	-17%	21	51

It is observed that the noise increases the number of iterations needed. In these experiments the Manhattan distance metric clearly provides better results than Euclidean distance. In this way, this metric has a better noise tolerance than the others.

## Discussion

The experiments are conducted with a heterogeneous set of 3D models. These models are composed of thousands of 3D points and they have been generated synthetically. The different experiments designed aims to reproduce a representative sample of the different scenarios where 3D rigid registration techniques are often used. In all the cases tested, the distance metrics analysed in this work reach the convergence. That is, all of them achieve the main objective of the algorithm and they are robust again registering partial data and noise in the model and in the data clouds.

In terms of accuracy, the three distance metrics have similar results. The cases with noise figures, the final error obtained is close to the mean of the magnitude of the introduced noise as expected.

The main focus of this study is the performance. The analytical computational cost shows that the low-cost distance metrics should have less time delay than the Euclidean one. In addition, they both are composed of primitive functions of the repertory of instruction of the architecture. The experiments performed on this matter, shows different results. The Chebyshev metric has shown a contradictory behaviour since a performance gain was expected instead of a loss. The Manhattan metric showed the anticipated behaviour regarding to its performance.

On the one hand, the proposal adapted to the Chebyshev metric has shown a constant execution time worsening along all the scenarios. This fact is due to the slower convergence because it provides bad distance approximations for the matching, and then more iterations are needed to complete the registration as shown in the convergence evolution figures. This means more execution time that is not balanced by the computational cost reduction in the distance computation itself. For this reason, the proposal that uses the Chebyshev distance is discarded, at least for its cost reduction properties.

On the other hand, the proposal implemented with the Manhattan metric has shown, in all cases, a better performance than the original implementation with the Euclidean one. The average execution time speedups obtained by this metric is about 14%. It is remarkable that the maximum improvement is produced not only by better time cost of the metric but also the lower iterations needed to reach convergence. It has been experimentally proven that the Manhattan metric offers better performances in the tested scenarios than the Euclidean one regarding execution time, and at the same time it keeps a similar registration quality. Therefore, the Manhattan metric, unlike Chebyshev one, becomes an alternative that should be considered for reducing the computational cost of the algorithm in general, taking into account that this improvement is boosted when we deal with a larger number of points, so its use is appropriate for high-resolution applications.

## Conclusions and Future Work

In this work, it is proposed an improvement for the ICP algorithm which is able to reduce its computational cost effectively for high resolution applications. This cost reduction affects the execution time of the algorithm, making it decrease in different scenarios. The research is focused on the distance metric used since this is one of the most frequent operator used by the algorithm. The performance improvement is due to the replacement of the classic Euclidean distance metric with other metric with less computational cost.

After the analysis and the exhaustive experimentation carried out in this work, some conclusions can be drawn about the results obtained. The case tested in this research concern specifically to 3D points, but similar findings should be drawn in others dimensions. In first place, it should be noted that different distance metrics can be used in the ICP algorithm to make the registration of point clouds. There exists many distance metrics with quite different formulations, but the main goal of this research is to find ways to reduce the computational cost of the ICP algorithm. So that, the work is specifically focused on low computational cost distance metrics. Among them it is chosen the Chebyshev and Manhattan distance metrics as candidates due to their simple mathematical formulation.

The experiments prove the convergence, accuracy, robustness and performance of the candidate metrics. The three former features are similar for all operators. However, the empirical work made demonstrate that a simpler mathematical formulation of the metric does not directly involves better performance. The speed of convergence (or number of iterations to reach the convergence) is the other aspect to take into account apart from its mathematical formulation. The experimentation made shows that the Manhattan distance metric is able to improve the Euclidean function in many scenarios, while the Chebyshev distance metric produces worst results. Thus, this last metric is discarded as candidate.

Finally, the proposed contribution can be extended or improved in several ways: the same concept of cost reduction could be applied in parallel architectures, both in CPU and GPU to explore both possibilities to increase performance. It is also possible to modify variants where the matching phase has not been altered, in order to apply this performance increase to other contributions that improve other parts of the algorithm. Furthermore, a custom low cost distance metric might be created to test its effect over the algorithm; or even a custom hardware architecture could be designed. This last option means a computational implementation of the algorithm, mainly the low-cost distance operator (the one that we have simulated here via software or a custom one), with the goal of reaching huge speedups.
